# Phase 2 Multicenter Study of Gantry-Based Stereotactic Radiotherapy Boost for Intermediate and High Risk Prostate Cancer (PROMETHEUS)

**DOI:** 10.3389/fonc.2019.00217

**Published:** 2019-04-02

**Authors:** David Pryor, Mark Sidhom, Sankar Arumugam, Joseph Bucci, Sarah Gallagher, Joanne Smart, Melissa Grand, Peter Greer, Sarah Keats, Lee Wilton, Jarad Martin

**Affiliations:** ^1^Princess Alexandra Hospital, Brisbane, QLD, Australia; ^2^Queensland University of Technology, Brisbane, QLD, Australia; ^3^Liverpool and Macarthur Cancer Therapy Centres, Sydney, NSW, Australia; ^4^University of New South Wales, Sydney, NSW, Australia; ^5^Ingham Institute, Sydney, NSW, Australia; ^6^St George Hospital, Cancer Care Centre, Sydney, NSW, Australia; ^7^Department of Radiation Oncology, Calvary Mater Newcastle Hospital, Newcastle, NSW, Australia; ^8^University of Newcastle, Newcastle, NSW, Australia

**Keywords:** stereotactic, radiation, boost, prostate cancer, linac

## Abstract

**Objectives:** To report feasibility, early toxicity, and PSA kinetics following gantry-based, stereotactic radiotherapy (SBRT) boost within a prospective, phase 2, multicenter study (PROMETHEUS: ACTRN12615000223538).

**Methods:** Patients were treated with gantry-based SBRT, 19–20 Gy in two fractions delivered 1 week apart, followed by conventionally fractionated IMRT (46 Gy in 23 fractions). The study mandated MRI fusion for RT planning, rectal displacement, and intrafraction image guidance. Toxicity was prospectively graded using the Common Terminology Criteria for Adverse Events version 4.0 (CTCAE v4).

**Results:** Between March 2014 and July 2018, 135 patients (76% intermediate, 24% high-risk) with a median age of 70 years (range 53–81) were treated across five centers. Short course (≤6 months) androgen deprivation therapy (ADT) was used in 36% and long course in 18%. Rectal displacement method was SpaceOAR in 59% and Rectafix in 41%. Forty-two and ninety-three patients were treated at the 19 Gy and 20 Gy dose levels, respectively. Median follow-up was 24 months. Acute grade 2 gastrointestinal (GI) and urinary toxicity occurred in 4.4 and 26.6% with no acute grade 3 toxicity. At 6, 12, 18, 24, and 36 months post-treatment the prevalence of late grade ≥2 gastrointestinal toxicity was 1.6, 3.7, 2.2, 0, and 0%, respectively, and the prevalence of late grade ≥2 urinary toxicity was 0.8, 11, 12, 7.1, and 6.3%, respectively. Three patients experienced grade 3 late toxicity at 12 to 18 months which subsequently resolved to grade 2 or less. For patients not receiving ADT the median PSA value pre-treatment was 7.6 ug/L (1.1–20) and at 12, 24, and 36 months post-treatment was 0.86, 0.36, and 0.20 ug/L.

**Conclusions:** Delivery of a gantry-based SBRT boost is feasible in a multicenter setting, is well-tolerated with low rates of early toxicity and is associated with promising PSA responses. A second transient peak in urinary toxicity was observed at 18 months which subsequently resolved. Follow-up is ongoing to document late toxicity, long-term patient reported outcomes, and tumor control with this approach.

## Introduction

When treating localized prostate cancer with definitive external beam radiotherapy (EBRT), multiple studies have demonstrated that dose escalation improves local control, freedom from biochemical failure, freedom from distant metastases, and reduces the need for salvage therapies, however, this has commonly come with a modest increase in genitourinary or gastrointestinal toxicity (1, 2). Brachytherapy has been established as an effective method to deliver high-dose boosts. The ASCENDE-RT trial demonstrated a marked reduction in biochemical relapse in men with unfavorable risk prostate cancer who underwent a low-dose-rate (LDR) boost compared to a conventional EBRT boost to 78Gy (3). However, brachytherapy is limited to a small number of specialized centers, and although it is a minimally invasive procedure, it requires short term hospitalization and anesthesia and can be associated with a higher risk of urinary toxicity, including urethral strictures and incontinence (4).

Stereotactic body radiotherapy (SBRT) offers a non-invasive alternative to deliver high doses per fraction. A number of studies have demonstrated the feasibility and tolerability of delivering SBRT boosts approximating high-dose-rate (HDR) brachytherapy schedules (18 to 20 Gy in 2–3 fractions) utilizing the dedicated CyberKnife platform (5–10). With the introduction of volumetric modulated arc therapy (VMAT), flattening filter free (FFF) delivery and refinements in intrafraction image guidance, high-dose SBRT boosts can now be efficiently delivered using the more widely available, gantry-based, linear accelerators.

We aimed to investigate the feasibility and tolerability of delivering a gantry-based, high-dose SBRT boost protocol in men with national comprehensive cancer network (NCCN) intermediate and high risk prostate cancer in an Australian multicenter setting. This report outlines the feasibility, early toxicity and PSA kinetics of this approach in the first 135 patients treated with a median follow-up of over 2 years.

## Methods

PROMETHEUS (PROstate Multicenter External beam radioTHErapy Using Stereotactic boost: ACTRN12615000223538) is a Phase 2, multicenter clinical trial evaluating a high-dose SBRT boost to the prostate in combination with fractionated external beam radiotherapy. Ethics approval for this study was granted by each participating institution's Human Research Ethics Committee, and written informed consent was obtained from all participants.

### Patient Eligibility

The study included men with a histological diagnosis of NCCN intermediate or high-risk prostate adenocarcinoma and ECOG performance status 0–1. Exclusion criteria included clinical T4 disease, nodal, or distant metastases, severe obstructive urinary symptoms requiring catheterization or transurethral resection prior to RT, prior pelvic radiotherapy, inflammatory bowel disease, hip prosthesis and inability to have an MRI or fiducial marker insertion.

### Treatment

**Figure 1:**
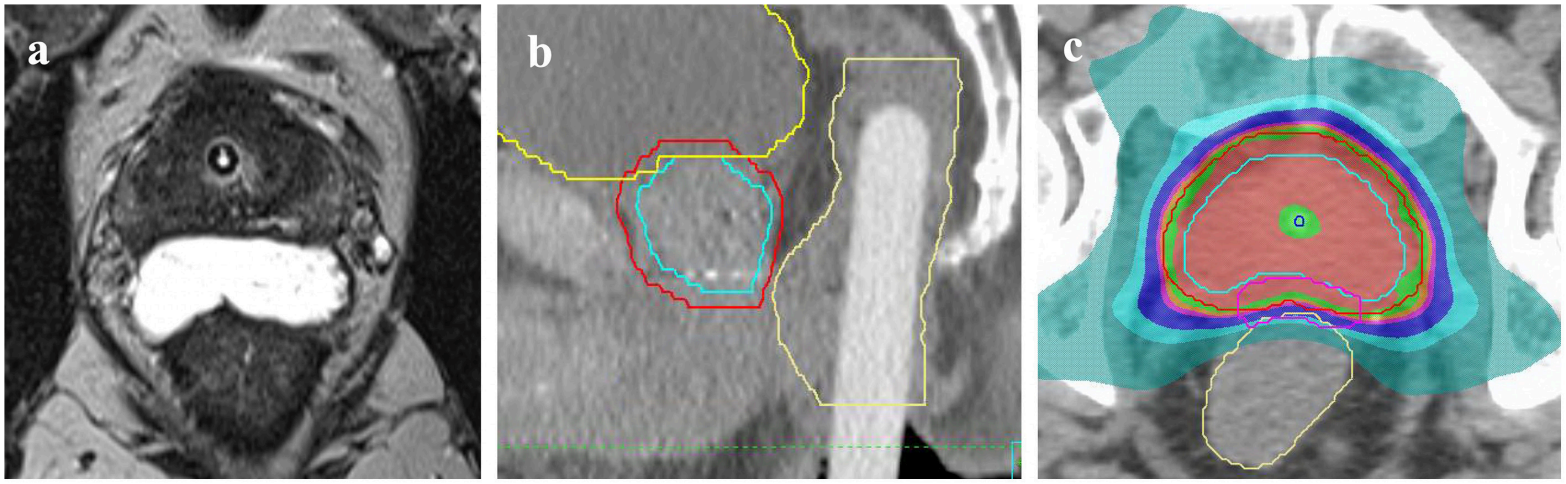
Methods of rectal displacement **(a)** SpaceOAR, **(b)** Rectafix. An example of the homogenous dosing employed **(c)** with red isodose representing 20 Gy, green 19 Gy, pink 18 Gy, dark blue 16 Gy, light blue 14 Gy, and aqua green 9.5 Gy. Contours represent prostate CTV (light blue outline), PTV (red) and rectum (yellow).

Use of a rectal displacement device (Figure 1) was mandatory with allowable options including SpaceOAR (Augmenix, Waltham, USA) or Rectafix (Scanflex Medical AB, Tumstocksvägen, Sweden). In the case of Rectafix this was used only for the SBRT fractions. A planning MRI, fiducial markers, and intrafraction image guidance were also mandatory. Patients were planned with a comfortably full bladder and empty rectum. The use of a rectal enema was encouraged prior to planning and SBRT treatments. An indwelling catheter (IDC) could be used to aid delineation of the urethra, but was not mandatory. The set-up for the primary planning CT was to be the same as for treatment (e.g., with or without IDC). Full technical details have been described previously (11).

The SBRT boost clinical target volume (CTV) incorporated the prostate plus any observed extracapsular or seminal vesicle (SV) extension as defined on MRI. The SBRT planning target volume (PTV) was an expansion of 5 mm, except posteriorly where it was 3 mm. Prescription dose for the SBRT boost commenced at 19 Gy in two fractions, 1 week apart, delivered using one to two coplanar VMAT arcs, with an option for increased dose rate via FFF. The protocol allowed for stepwise dose escalation by 1 Gy (to 20 Gy) when 20 patients reached 12 months follow-up with <15% incidence of severe genitourinary or gastrointestinal toxicity and no grade 4 toxicity.

Dose specifications and constraints are outlined in Table 1. Image guidance comprised an initial fiducial marker match and verification of soft tissue anatomy with cone beam computed tomography (CBCT). Intra-fraction image guidance was achieved using ExacTrac^®^ (Brainlab AG, Germany), triggered kV imaging (Varian medical systems, Palo Alto, USA), mid-treatment CBCT or an in-house real-time continuous kilovoltage fiducial marker tracking software (SeedTracker) (12).

**Table 1:** PROMETHEUS SBRT dose constraints.

**Constraint**	**Per-protocol**	**Minor variation**	**Major variation**
CTVsbrt D98^a^	>100% TD	95–100% TD	<95% TD
PTVsbrt D50^a^	<105% TD	105–110% TD	>110% TD
PTVsbrt D90^a^	>100% TD	95–100% TD	<95% TD
PTVsbrt D95^a^	>95% TD	90–95% TD	<90% TD
PTVsbrt D99^a^	>16 Gy	15–16 Gy	<15 Gy
PTVsbrt Dmax to 0.1 cc	<110% TD	110–120% TD	>120% TD
Rectal wall Dmax to 0.1 cc	<17 Gy	17–17.5 Gy	>17.5 Gy
Rectal wall V16 Gy	<0.5 cc	0.5–1 cc	>1 cc
Rectal wall V14 Gy	<3 cc	3–5 cc	>5 cc
Rectal wall V10 Gy	<40%	40–50%	>50%
Rectum posterior wall	<8.5 Gy	8.5–9.5 Gy	>9.5 Gy
Bladder Dmax to 0.1 cc	<110% TD	110–120% TD	>120% TD
Bladder V19 Gy	<10 cc	10–15 cc	>15 cc
Bladder V17 Gy	<15%	15–20%	>20%
Bladder V9 Gy	<50%	50–60%	>60%
Urethra PRV Dmax to 0.1 cc	<110% TD	110–115% TD	>115% TD
Urethra PRV V105% TD	<5%	5–15%	>15%
Femoral neck Dmax to 0.1 cc	<8 Gy	8–9 Gy	9 Gy
Penile bulb Dmax to 0.1 cc (recommended)	100% TD	100–105% TD	>105% TD
Penile bulb V10 Gy (recommended)	<3cc	3–5 cc	>5 cc
Intermediate dose spillage: ratio of volumes receiving 50% TD to 100% TD	<4	4–5	>5
High dose conformation: V100% TD/PTVsbrt volume	<1.1	1.1–1.2	>1.2
Total monitor units	<3 × Dose in cGy	3–3.5 × Dose in cGy	>3.5 × Dose in cGy

*TD, Target dose (19 or 20 Gy)*.

a *these volumes may exclude the urethra PRV*.

The EBRT component commenced 2 weeks following the SBRT boost. The CTV included the prostate and at least the proximal 1 cm of SV for intermediate risk disease, proximal 2 cm for high risk disease and entire seminal vesicles in the case of cT3b disease. Elective pelvic nodal irradiation was recommended, but not mandated, if the estimated risk of nodal involvement was >15% according to the MSKCC nomogram. Pelvic nodal groups were contoured as per the Radiation Therapy Oncology Group (RTOG) guidelines. CTV to PTV expansion was 5 to 7 mm. A dose of 46 Gy in 23 daily fractions was prescribed using IMRT or VMAT with daily image guidance. Dose constraints for the EBRT component have been outlined previously (11). Following publication of the PROFIT and CHHIP hypofractionation studies, the protocol was amended in 2017 to allow an alternate EBRT fractionation schedule of 36 Gy in 12 daily fractions (13–15).

The type and duration of androgen deprivation therapy (ADT) was at investigators discretion; however, 6 months of ADT was recommended for unfavorable intermediate risk or lower tier high risk disease (one high risk factor), and 18–24 months for men with multiple high risk factors.

### Quality Assurance

Each center was required to complete a benchmarking contouring and planning case and submit the first three plans at each dose level for external, real-time, pre-treatment review by one of the lead investigators. Following this any plan recording a major dosimetric variation or more than three minor variations was also required to be externally reviewed prior to treatment commencing.

#### Assessments

Prostate specific antigen (PSA) value and toxicity assessments using common terminology criteria for adverse events version 4 (CTCAE v4) were collected pre-treatment, at the completion of treatment, 6 weeks post-treatment then six monthly thereafter up to 5 years.

### Endpoints and Statistical Analysis

The primary objectives of the study were to determine the feasibility and safety of the SBRT boost protocol in a multicenter setting. Multicenter recruitment and deliverability was considered feasible if three or more centers contributed five or more patients to the study without major violations. A cumulative incidence of >15% grade 3 gastrointestinal or genitourinary toxicity (excluding erectile dysfunction) would require halting the protocol. A prevalence of late grade ≥2 gastrointestinal or genitourinary toxicity of <15% over the 5 years of follow-up was considered acceptable. Toxicity events occurring within 6 months were scored as acute and events occurring 6 months and beyond were considered late toxicity. Secondary endpoints included patient reported outcomes (EPIC 26) and efficacy, assessed via biochemical control after 3 years using the Phoenix definition of PSA nadir + 2 (16).

### Results

Between March 2014 and July 2018, 135 patients underwent protocol treatment across five centers demonstrating feasibility of recruitment and delivery in a multicenter setting. An additional 11 patients were screened for the study but did not proceed with protocol treatment. Nine patients were excluded from the study due to either an inability to undergo gold seed placement (*n* = 2), an inability to tolerate rectafix (*n* = 2), an inability to achieve protocol constraints due to inadequate rectal separation with SpaceOAR (*n* = 3) or Rectafix (*n* = 1) or due to small bowel abutting the boost CTV (*n* = 1). One patient withdrew, proceeding to surgery instead, and one patient was withdrawn from the study prior to treatment due to worsening obstructive urinary symptoms.

Median follow-up was 24 months. Patient characteristics are outlined in Table 2.

**Table 2:** Patient characteristics (*n* = 135).

**Characteristics**	
**AGE IN YEARS**	
Median (range)	70 (53–81)
**RISK CATEGORY**	
Intermediate	103 (76%)
High	32 (24%)
**ANDROGEN DEPRIVATION**	
Nil	62 (46%)
≤6 months	50 (37%)
>6 months	23 (17%)
**RECTAL SEPARATION**	
SpaceOAR	80 (59%)
Rectafix	55 (41%)
**SBRT DOSE LEVEL**	
19Gy	42 (31%)
20Gy	93 (69%)
**EBRT Dose Schedule**	
46Gy in 23#	127 (94%)
36Gy in 12#	8 (6%)
**ELECTIVE PELVIC EBRT**	
No	124 (92%)
Yes	11 (8%)

### Rectal Displacement

The majority of patients (59%) underwent insertion of SpaceOAR to achieve rectal displacement. Moderate and severe discomfort associated with SpaceOAR was reported by 3.8 and 2.5% of men, respectively. The Rectafix device was used in the remainder (41%) to achieve rectal displacement for the SBRT fractions. Moderate and severe discomfort with Rectafix was reported by 35 and 14% of men, respectively.

### Acute Toxicity

Physician reported (CTCAE v4) acute grade 2 gastrointestinal toxicity occurred in six patients (4.4%) with no acute grade 3 toxicity reported. Acute grade 2 urinary toxicity occurred in 36 patients (26.6%) with no acute grade 3 toxicity reported.

### Late Toxicity

**Figure 2:**
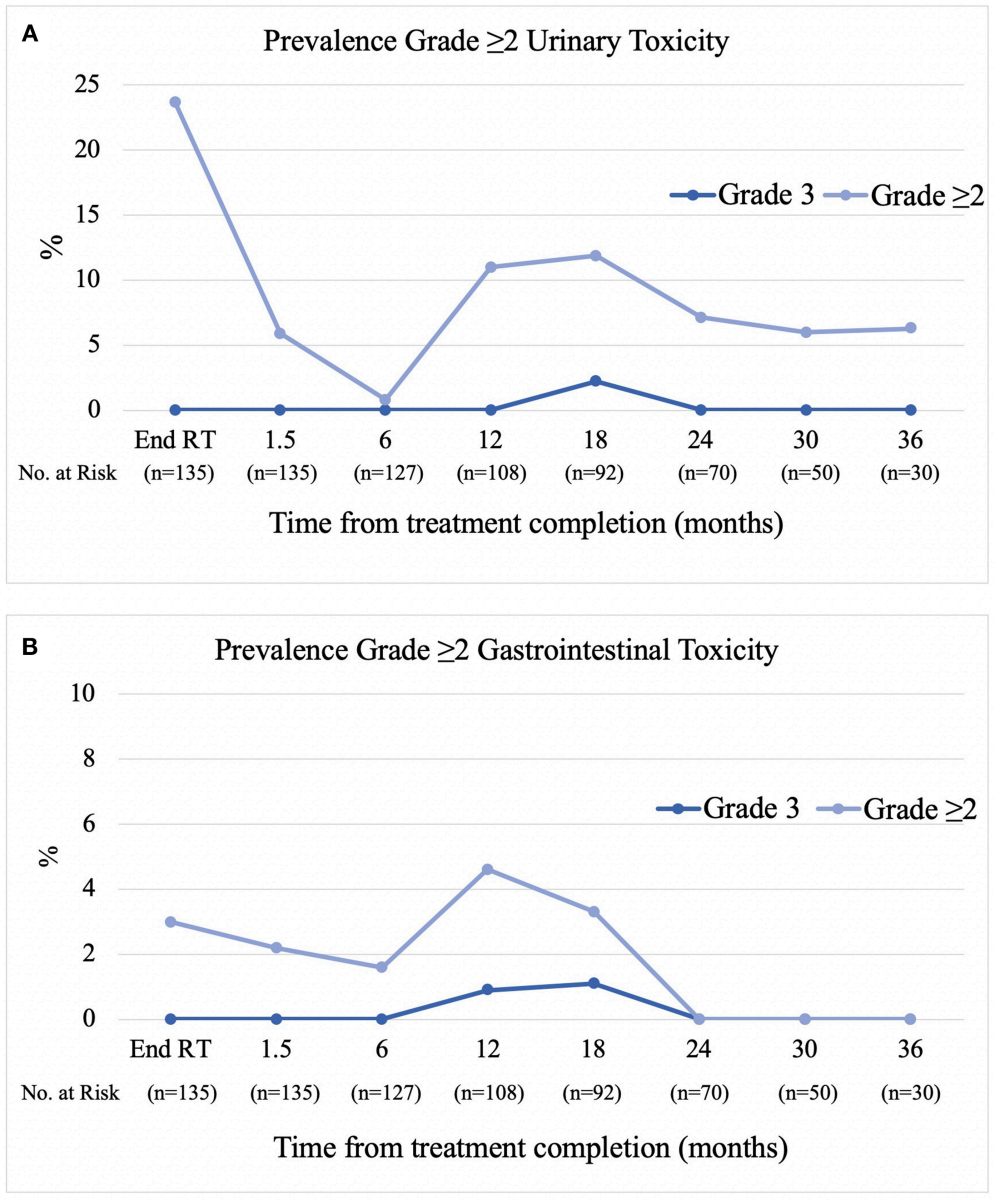
Prevalence of CTCAE v4 Grade ≥2 Urinary **(A)** and Gastrointestinal **(B)** Toxicity.

The prevalence of physician reported (CTCAE v4) late grade ≥2 gastrointestinal toxicity at 6, 12, 18, 24, and 36 months post-treatment was 1.6, 3.7, 2.2, 0, and 0% respectively (Figure 2A). The predominant type of toxicity was proctitis or proctalgia with only two grade 2 rectal bleeding events. The cumulative incidence of late grade ≥2 and grade 3 gastrointestinal toxicity was 4.5 and 2%, respectively. The prevalence of late grade ≥2 urinary toxicity at 6, 12, 18, 24, and 36 months was 0.8, 11, 12, 7.1, and 6.3%, respectively (Figure 2B). The cumulative incidence of late grade ≥2 and grade 3 urinary toxicity was 24.9 and 2.2%, respectively. Overall, three patients have experienced grade 3 toxicity. The first patient experienced a combination of grade 3 proctitis, cystitis, urinary incontinence, and pelvic pain peaking at 18 months post-treatment. He underwent hyperbaric oxygen (HBO) treatment with symptoms completely resolving by the 24 month follow-up. The second patient experienced grade 3 cystitis with haematuria and clot retention at 18 months and underwent HBO in addition to cystoscopy and diathermy of telangiectasias in the prostatic urethra. The third patient experienced grade 3 proctitis at 12 months and underwent HBO treatment with proctitis resolving to grade 1 by the 18 month follow-up.

### PSA Response

**Figure 3:**
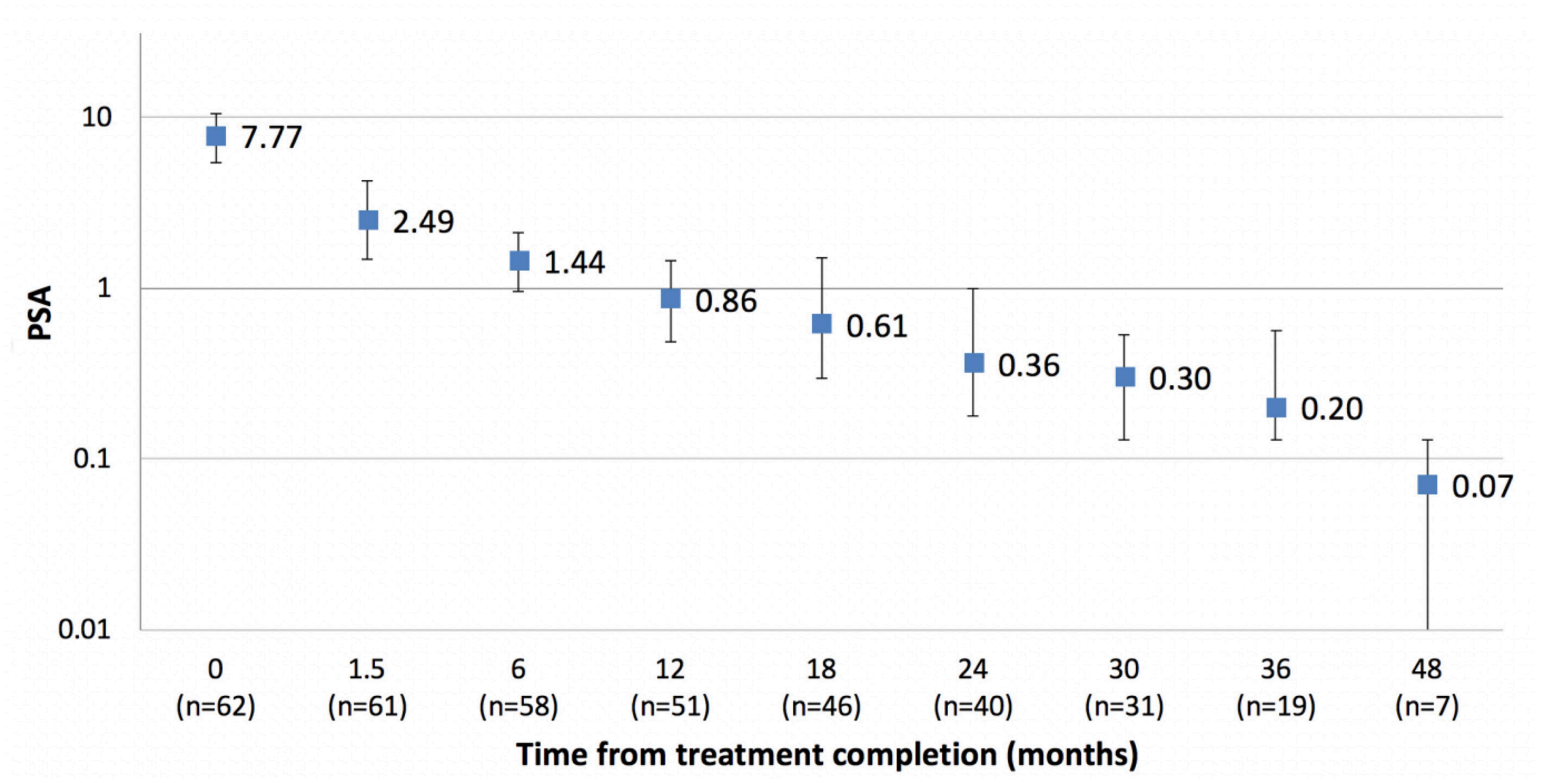
Median PSA response (with interquartile range) in cohort treated without ADT (*n* = 62).

The median PSA value pre-treatment for the entire cohort was 8.9 ug/L (1.1–63 ug/L) and at 12, 24, and 36 months post-treatment was 0.5, 0.27, and 0.21 ug/L, respectively. For the cohort not receiving ADT the median PSA value pre-treatment was 7.6 ug/L (1.1–20 ug/L) and at 12, 24, and 36 months post-treatment was 0.86, 0.36, and 0.20 ug/L, respectively (Figure 3). Two patients have experienced BCF to date. Both had high risk disease and received ADT as part of their initial treatment. One patient experienced a rapid PSA rise with demonstrable bone metastases at 20 months, the other a PSA relapse at 33 months. The 2 year freedom from biochemical recurrence was 98.6%.

## Discussion

We have demonstrated that our gantry-based SBRT prostate boost protocol, delivering 19–20 Gy in 2 fractions, is feasible in a multicenter study, is well tolerated with low rates of early toxicity and is associated with promising PSA responses. This is in keeping with previous studies evaluating SBRT boosts, predominantly utilizing the Cyberknife platform (5–10, 17). A summary of these studies is provided in Table 3 with all reporting low rates of grade ≥3 toxicity.

**Table 3:** Summary of studies evaluating SBRT boost schedules.

**Author**	**Year**	***N***	**Risk**	**Median F/U months**	**Conventional dose**	**Pelvic nodal RT**	**ADT use**	**Boost dose**	**Platform**	**Biochemical control**	**Late toxicity grade ≥3**
Miralbell et al. (18)	2010	50	L/I/H	63	64 Gy/32#	56%	66%	10–16 Gy/2#	Linac	5 year 98%	GU 0% GI 10% ^*^
Katz and Kang (6)	2010	73	I/H	33	45 Gy/25#	Yes	49%	19–21 Gy/3#	CK	3 year 89.5% (I) 77.7% (H)	GU 1.4% GI 0% ^*^
Lin et al. (9)	2014	41	H	42	45 Gy/25#	Yes	100%	21 Gy/3#	CK	4 year 91.9%	GU 0% GI 0% ^∧^
Mercado et al. (5)Paydar et al. (17)	2016	108	I/H	53	45–50.4 Gy/25–28#	No	64%	19.5 Gy/3#	CK	3 year 100% (I) 89.8% (H)	GU 6% GI 1% ^∧^
Anwar et al. (7)	2016	48	I/H	43	45–50 Gy/25#	Yes	93%	19–21 Gy/2#	CK	3 year 95% 5 year 90%	GU 2% GI 0% ^∧^
Kim et al. (8)	2017	39	I/H	54	45 Gy/25#	Yes	Nil	21 Gy/3#	CK	5 year 94.7%	GU 0% GI 0% ^*^
Pasquier et al. (10)	2017	76	I	26	46 Gy/23#	No	Nil	18 Gy/3#	CK (60) Linac (16)	2 year 98.7%	GU 0% GI 1.3% ^∧^
PROMETHEUS	2019	135	I/H	24	46 Gy/23# 36 Gy/12#	8%	54%	19–20 Gy/2#	Linac	2 year 98.6%	GU 2% GI 2% ^∧^

N, number of patients; L, Low risk; I, Intermediate risk; H, High risk; F/U, follow-up; RT, radiation therapy; #, fractions; ADT, androgen deprivation therapy; ^∧^, Toxicity graded using CTCAE criteria;

* *, Toxicity graded using RTOG criteria*.

The very low rate of rectal toxicity in our study is likely attributable to the use of rectal displacement in combination with rapid coplanar (predominantly FFF) VMAT delivery with intra-fraction positional verification and 3 mm posterior margins. There was a small peak in GI events at the 12–18 month mark (3.7% grade ≥2) with a predominance of proctalgia and urgency rather than the classical proctitis picture of frequency, mucous discharge, or bleeding. This may reflect more of a self-limiting, peripheral neuropathy rather than mucosal effects. Regarding the method of rectal displacement we have previously reported that SpaceOAR and Rectafix provide similar dosimetric advantages in terms of rectal sparing (19). SpaceOAR was associated with less discomfort and has the added advantage of being *in situ* for the fractionated component of treatment; however, the additional cost is not routinely covered in Australia currently and there is a small risk of inadequate separation. Rectafix has the advantage of being inexpensive to implement and is not dependent on good tissue planes to achieve improvements in rectal sparing.

Despite delivering an equivalent dose in 2 Gy fractions (EQD2) of ~100–110 Gy (for an α/β ratio of 3 and 1.5, respectively), the urinary toxicity profile was also acceptable with no grade 3 acute toxicity and only two late grade 3 events to date. The prevalence of grade ≥2 urinary toxicity at 2–3 years post treatment was in the order of 6–7%. In our study we specified urethral constraints to avoid high dose dumping on the urethra, mandated MRI fusion for contouring in addition to intrafraction verification with 3–5 mm PTV margins to reduce the volume of bladder neck, membranous urethra, and penile bulb receiving high dose levels.

Similar to that previously reported by Collins et al. we observed a biphasic pattern of urinary toxicity (20). The highest prevalence of grade ≥2 urinary toxicity was seen at the end of treatment which settled in the months following treatment and was then followed by a second transient peak (12%) at the 12–18 month mark, dominated by a cystourethritis picture (urgency, dysuria, and frequency). Beyond 18 months the prevalence decreased again to between 6 and 7%. As previously reported, this transient subacute flare of cystourethritis can respond to alpha-blockers and anti-inflammatories with the majority of patients recovering to near baseline without the need for invasive procedures (21). Two patients experienced grade 3 urinary toxicity, the first in combination with grade 3 GI toxicity and pelvic pain which completely resolved after a course of HBO and the second resolving spontaneously. It is unknown whether the former would have self-resolved without HBO.

Although follow-up is too short to assess biochemical control endpoints, the median PSA nadir in the cohort not receiving ADT is very low, suggesting there may not be a significant benefit in escalating above the current 20 Gy in 2 fraction dose level. The low PSA nadirs in this study are similar to that reported in previous studies utilizing SBRT boosts and not dissimilar to that reported for brachytherapy (6, 7, 9, 22, 23).

A gantry-based linac solution to deliver SBRT boosts offers a number of potential practical advantages. Gantry-based linacs are widely accessible and offer a non-invasive, convenient treatment which can be delivered in two to three outpatient procedures of 15–20 min each. Quality assurance can be automated and performed in real time, reducing the learning curve within centers and clinical networks. Technical sub-studies have validated the real time SBRT radiation dose delivery and treatment accuracy (24, 25). We have shown that a protocol incorporating rectal displacement devices, MRI fusion, margin reduction coupled with intrafraction image guidance and homogenous urethral dosing is associated with a low risk of significant urinary and rectal toxicity with a median follow-up of 24 months.

The ASCENDE-RT study has highlighted the importance of radiation dose for NCCN intermediate and high risk disease with a halving of biochemical relapse seen in the brachytherapy boost cohort (3). In a sequential SBRT dose escalation study (from 32.5 to 40 Gy in 5 fractions) Zelefsky et al. demonstrated that higher SBRT dose levels were associated with lower rates of PSA failure and positive post-treatment biopsy, with a modest increase in transient grade 2 urinary toxicity (26). In the setting of NCCN high risk disease, a number of non-randomized studies have recently reported promising results utilizing SBRT monotherapy or SBRT boost, providing a rationale for randomized studies of SBRT in this cohort (6, 7, 9, 27). A large randomized study comparing brachytherapy boost with SBRT boost would be required to evaluate the relative efficacy and toxicity of these two approaches; however, to our knowledge no phase 3 trials are underway. Two studies commencing recruitment in 2019 will be exploring SBRT dose fractionation questions specifically in the unfavorable intermediate and lower-tier high risk groups. The new arm of the PACE study (NCT 01584258), cohort “C,” will compare conventional EBRT (78 Gy in 39 fractions or 62 Gy in 20 fractions) to an SBRT schedule delivering 36.25 Gy in 5 fractions to the PTV and 40 Gy in 5 fractions to the CTV. NINJA (TROG 18.01, ACTRN 12618001806257) is an extension of the current study and will compare our SBRT boost protocol (20 Gy in 2 fractions followed by 36 Gy in 12 fractions) to an SBRT monotherapy protocol delivering 36.25 Gy to the PTV and 40 Gy to the CTV in 5 fractions.

Follow-up is ongoing to enable reporting of longer term late toxicity, patient reported outcomes and biochemical control.

## Conclusion

Delivery of gantry-based SBRT prostatic boost is feasible and well tolerated with low rates of early toxicity and promising PSA responses. A second transient peak in genitourinary toxicity was observed at 18 months which subsequently resolved. Follow-up is ongoing to document late toxicity, long term patient reported outcomes and tumor control with this approach. A randomized study comparing this regimen to SBRT monotherapy has also commenced.

## Data Availability

The datasets generated for this study are available on request to the corresponding author.

## Author Contributions

JM and MS developed the study concept and drafted the manuscript. DP drafted and revised the manuscript. SA, PG, JS, SK, SG, MG, JB, and LW contributed to the protocol development, study administration, data analysis and manuscript revision. All authors were involved in the running of the study and the revision and approval of the final manuscript.

### Conflict of Interest Statement

DP has participated in an Advisory Board for Ipsen. JM has received research funding from mundipharma, and participated in Advisory Boards for Ferring pharmaceuticals and Janssen. The remaining authors declare that the research was conducted in the absence of any commercial or financial relationships that could be construed as a potential conflict of interest.
